# The Prevalence and Risk Factors of Plantar Fasciitis Amongst the Population of Jazan

**DOI:** 10.7759/cureus.29434

**Published:** 2022-09-21

**Authors:** Zenat Khired, Muhannad H Najmi, Ahmed A Akkur, Mashhour A Mashhour, Khalid A Bakri

**Affiliations:** 1 Surgery, Jazan University, Jazan, SAU; 2 Medicine and Surgery, Jazan University, Jazan, SAU

**Keywords:** jazan region, jazan city, heel pain, population, plantar fasciitis, risk factors, incidence

## Abstract

Background

Plantar fasciitis develops as a consequence of irritation of the plantar fascia, which is responsible for supporting the arches and absorbing shock. Multiple factors can contribute to plantar fasciitis, but the most common factor is overuse stress. The classical presentation is a sharp pain that is felt at the plantar aponeurosis (near the area of its insertion on the medial process of the calcaneal tuberosity), and it is possible to find a heel spur (osteophyte) in some cases. Most treatments for plantar fasciitis are ineffective and unsatisfactory for patients.

Objective

To estimate the prevalence and assess risk factors for plantar fasciitis among the population of the Jazan region.

Methods

A cross-sectional online survey was conducted on the population of the Jazan region. An online Google form questionnaire was prepared and distributed to the study population. Data was then entered and analyzed using IBM SPSS (Statistical Package for the Social Sciences) Statistics, version 21.0.

Results

A total of 695 participants were enrolled in the study. Out of that, 350 (50.4%) of the participants were males, while 345 (49.6%) were females. About the age of the participants, 507 (72.9%) were younger than 40 years of age. Participants with hypertension were 43 (6.2%), and 37 (5.3%) participants had diabetes. In terms of occupation, 120 (17.3%) worked in healthcare, 187 (26.9%) taught, and 43 (6.2%) served in the military. A great quantity of standing or walking was necessary for the jobs for 127 people (18.3%), while moderate amounts were recorded for 273 people (39.3%). The most commonly reported lower limb conditions were: pes planus (low arches) in 26 (3.7%) participants; and weakness of the gastrocnemius, soleus, and intrinsic foot muscles. The most commonly reported exercises were walking for 499 (71.8%) participants and jogging for 97 (14%) participants. The prevalence of plantar fasciitis was found to be 37% of the participants. Regarding the Foot and Ankle Outcome Score (FAOS) survey, the mean symptoms subscale score was 57.81 + 11.28, the mean pain subscale score was 72.87±20.84, the mean daily living subscale score was 72.73 ± 22.25, the mean sports and recreation subscale score was 76.83 ± 23.06, and the mean quality of life subscale score was 70.23 ± 25.17. Multivariate logistic regression was done and the following factors predicted a higher rate of plantar fasciitis: being 40 to 55 years old (p < 0.001, odds ratio = 2.15), being 56 to 65 years old (p = 0.037, odds ratio = 3.58), being obese (p = 0.031, odds ratio = 2.16), having weakness of the gastrocnemius, soleus, and the intrinsic foot muscles (p = 0.003, odds ratio = 7.39), jobs requiring a great amount of time standing or walking (p < 0.001, odds ratio = 3.17), and jobs requiring a moderate amount of time standing or walking (p = 0.012, odds ratio = 1.83). Being male predicted a lower rate of plantar fasciitis (p < 0.001, odds ratio = 0.52).

Conclusion

Plantar fasciitis is a prevalent and disabling condition with considerable effects on quality of life. Jobs that require long hours of walking or standing were associated with an increased risk of developing plantar fasciitis. Middle age, prolonged exercise, and gastrocnemius muscle tightness were also associated with plantar fasciitis. Efforts should be directed towards health education of the population regarding the risk factors and management of plantar fasciitis.

## Introduction

Plantar fasciitis occurs as a result of degenerative irritation of the plantar fascia that originates at the medial calcaneal tuberosity of the heel (as well as the surrounding perifascial structures). There are three segments of the plantar fascia, all of which arise from the calcaneus, and each plays an important role in normal foot biomechanics. It is important to recognize that the fascia is responsible for supporting the arch and absorbing shock. It is notable that despite the segment "itis," there are no inflammatory cells present in this condition [1,2,3]. Several factors can contribute to plantar fasciitis, but overuse stress is the most common cause. A sharp pain felt at the heel is the classic presentation, and it is possible to find a heel spur in some cases. Most treatments for plantar fasciitis are ineffective and dissatisfactory for patients [4].

The plantar fascia can be torn as a result of repetitive strain or trauma, among other factors, resulting in an overuse injury. In addition to pes planus, pes cavus, and limited ankle dorsiflexion, excessive pronation and supination may also contribute to plantar fasciitis. As a result of a pes planus, the plantar fascia is subjected to increased strain. Because the foot does not evert or absorb shock effectively, pes cavus can cause excessive strain on the heel. Patients with pes cavus may also experience tightness in the gastrocnemius, soleus, and/or other posterior leg muscles. The biomechanics of ambulation can be altered by these tight muscles. In about half of the cases, heel spurs also occur, although they are not directly responsible for this condition. The condition is frequently associated with runners and older adults, but obesity, heel pad atrophy, aging, and occupations that require prolonged standing can also be risk factors. Seronegative spondyloarthropathies have been found to have a relationship with plantar fasciitis, but systemic factors are unknown in approximately 85% of cases [5,6].

Plantar fasciitis is the most common cause of heel pain presented to outpatient clinics. While we are not aware of the exact incidence and prevalence of plantar fasciitis by age, estimates suggest that more than one million doctor visits occur annually because of plantar fasciitis [7,8].

The patient doesn't need to undergo imaging for plantar fasciitis to be diagnosed. However, X-rays or ultrasounds may be considered if the patient's history and physical examination indicate other injuries or conditions, or if the patient does not improve after a reasonable amount of time. These examinations might reveal that the inferior aspect of the heel has heel spurs or calcifications in the soft tissues. Furthermore, ultrasound may reveal thickening and swelling of the plantar fascia, which are typical signs of the condition. The provider may consider ordering an MRI to determine if the patient is suffering from tears, stress fractures, or osteochondral defects if conservative therapy fails to help the patient after longer periods of time [9,10].

Based on the level of pain, it is recommended to prescribe relative rest from aggravating activities. Pain can be relieved with ice after exercise, as well as with oral or topical non-steroidal anti-inflammatory drugs (NSAIDs). It has been demonstrated that deep friction massage of the arch and insertion is beneficial. Additionally, shoe inserts or orthotics and night splints can also be prescribed. The Achilles tendon, plantar fascia, gastrocnemius, and soleus muscles need to be stretched and rehabilitated properly by providers. If conservative procedures fail to relieve the pain, more sophisticated or invasive methods might also be considered, such as extracorporeal shock-wave therapy, botulinum toxin A, or different injections, including autologous platelet-rich plasma, dex prolotherapy, or steroids. Conservative therapy should, nonetheless, be used in conjunction with more sophisticated and invasive procedures. If this condition has become chronic and less intrusive treatments have failed, surgery should be the last resort [11-13].

A multidisciplinary approach to treating plantar fasciitis is required since no one treatment is universally effective. Even when a treatment is effective, symptoms frequently take weeks or months to go away. Many people, usually young people and athletes, suffer from plantar fasciitis. If the ailment is not treated properly, it may become incapacitating. Physical therapists, pharmacists, nurses, and rehabilitation specialists play a pivotal role in preventing the recurrence of symptoms, but patient education is also crucial. Patients must be informed that it may take weeks or months for their symptoms to go away. The patient might also be required to wear a night splint and enroll in a physical therapy program, and a podiatrist consultation could be necessary in order to obtain the proper footwear with sufficient arch support. Patients must be instructed on how to perform simple workouts at home to stretch their plantar fascia and be informed about the importance of stretching before beginning an exercise regimen and also about the benefit of losing weight. They should also receive education about the risks of prolonged standing. Acutely ill patients should be advised to refrain from going barefoot and to reduce repetitive heel-damaging workouts. The last recourse is a referral to an orthopedic surgeon if all of these techniques are unsuccessful [14-16].

The objectives of this study were to determine the prevalence and risk factors of plantar fasciitis among the population of the Jazan region.

## Materials and methods

A cross-sectional online questionnaire-based survey was conducted in the Jazan area in southwest Saudi Arabia with around 1.5 million people. This study targeted all adult male and female residents in the Jazan region. We excluded populations with a history of hip, knee, foot, or ankle surgery or stress fractures of the calcaneus, pregnant women (because of changes in weight and potential pedal oedema which may result in heel pain), and populations with medical conditions such as ankylosing spondylitis, systemic lupus erythematous, peripheral neuropathy, Sever’s disease, and tarsal tunnel syndrome.

A sample of 480 participants over the age of 15 years was selected. The sample size was calculated using the formula for a cross-sectional study using the following parameters: N = 1,201,118, p = prevalence of knowledge 50%, Z = 95% confidence interval, d=error ≤5%, and a 25% non-response rate. The study included an adult population who were willing to participate and who had pain that was localised to the medial aspect of the heel, regardless of the duration and onset of their pain and the presence or absence of restriction of ankle and foot movement.

A Google forum questionnaire was prepared, distributed throughout social media to the study population, and verified, and the questionnaire data were entered into the Statistical Product and Service Solutions (SPSS®) software for Windows® ver.21 (SPSS Inc, Chicago, IL, USA). The analysis involved descriptive statistics and also inferential statistics, depending on the purpose of each relationship. Normally, distribution data is managed by tests appropriate for this type of data, e.g., t-test and Chi-square tests were used to assess some associations between the study variables.

All the necessary official permissions were obtained before data collection. All participants were informed about the objectives of the study. Their consent to participate was requested. Ethical approval was received from the Standing Committee for Scientific Research-Jazan University (HAPO-10-Z-001), Reference No.: REC-43/06/140.

The pilot study was conducted among a small group of participants to assess the time that was taken for the respondents to fill up the questionnaire, understand and interpret the questions, and assess the validity. The feedback of the participants was taken into consideration in the final survey format, and the changes included correcting grammar and language mistakes, summarising and shortening long questions, and omitting some questions because of repetition. The pilot study participants were excluded from the main study.

## Results

A total of 695 participants were included in the study. Table 1 displays the socio-demographic profiles of the participants. With regards to participant age groups, 507 (72.9%) of the participants were less than 40 years old; 173 (24.9%) were between 40 and 55 years of age; 13 (1.9%) were 56 to 65 years old, and two (0.3%) were older than 65 years. As for the gender split, 350 (50.4%) of the sample group were males, while 345 (49.6%) were females. As for marital status, 299 (43%) of the participants were single, 375 (54%) were married, and 21 (3%) were divorced or widowed. With regards to educational qualifications, 571 (82.2%) of the participants had university-level education; 103 (14.8%) had high school education; 15 (2.2%) had intermediate school education, and six (0.9%) participants were uneducated. Regarding their employment status, 382 (55%) of the participants were employed, 85 (12.2%) were unemployed, and 228 (32.8%) had other jobs.

**Table 1:** Socio-demographic profile of the participants ( n= 695)

Demographical characteristics		n	%
Age			
Less than 40 years		507	72.90
40 - 55 years		173	24.90
56 - 65 years		13	1.90
Older than 65 years		2	0.30
Gender			
Male		350	50.40
Female		345	49.60
Marital status			
Single		299	43.00
Married		375	54.00
Divorced/widowed		21	3.00
Education			
University-level education		571	82.20
High school		103	14.80
Intermediate school		15	2.20
Uneducated		6	0.90
Employment status			
Employee		382	55.00
Unemployed		85	12.20
Others		228	32.80

**Figure 1:**
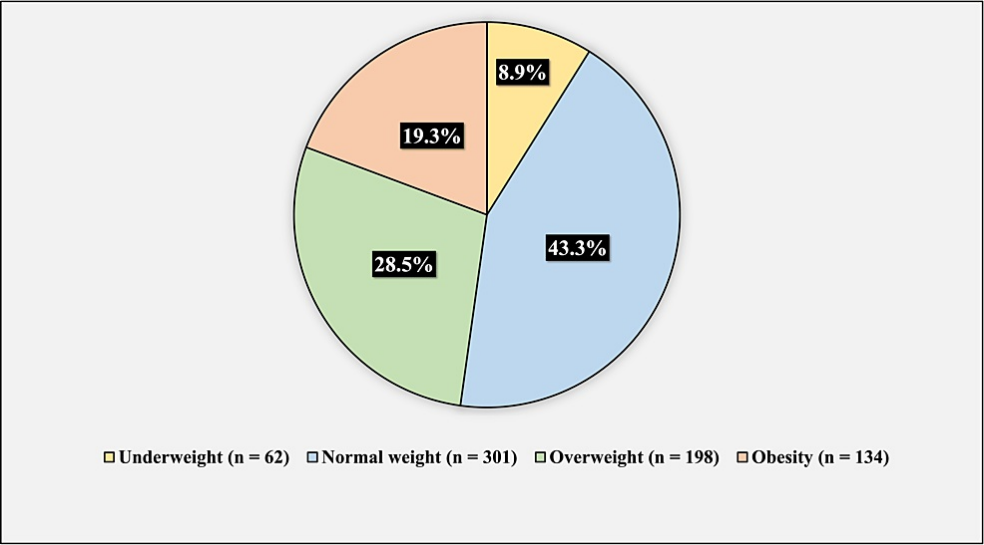
BMI of the participants The figure has been created by the authors.

Figure 1 shows the body mass index (BMI) of the participants. 62 (8.9%) of the participants were underweight, 301 (43.3%) had normal weight, 198 (28.5%) were overweight, and 134 (19.3%) participants were obese.

**Figure 2:**
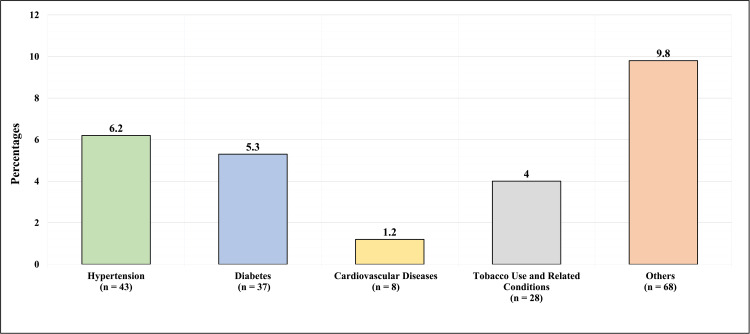
Comorbidities of the participants The figure has been created by the authors.

Figure 2 presents the comorbidities of the participants. Out of the total number of participants, 43 (6.2%) had hypertension, 37 (5.3%) had diabetes, eight (1.2%) participants had cardiovascular disease, 28 (4%) reported tobacco use or had tobacco-related conditions, while 68 (9.8%) had other comorbidities.

Table 2 demonstrates the job profile of the participants. With regards to the field of work, 120 (17.3%) were working in the healthcare sector, 187 (26.9%) were teachers, 43 (6.2%) were working in the military, 6 (0.9%) were bankers, 3 (0.4%) were in sales, and 316 (45.5%) worked in other fields. Concerning the amount of standing or walking that is required in their jobs, 127 (18.3%) reported that their work required a great amount of standing or walking, and 273 (39.3%) reported that their work required moderate amounts of the same. Additionally, 138 (19.9%) of the participants reported that their work required a slight amount of standing or walking, while 157 (22.6%) reported their work required no amount of standing or walking. The hours spent per day standing or sitting have been displayed respectively in Table 2.

**Table 2:** Job profile of the participants & the information on the walking, standing, and sitting time required in their jobs

Job profile of the participants (n = 695)		
Question	n	%
Field of work		
Healthcare	120	17.3
Teaching	187	26.9
Military	43	6.2
Banker	6	0.9
Job in a restaurant	3	0.4
Seller in the shop	20	2.9
Other	316	45.5
How much standing or walking is required from you in your job?		
Great amount	127	18.3
Moderate amount	273	39.3
A little amount	138	19.9
None	157	22.6
How many hours per day of standing or walking is required from you in your job?		
Less than 3 hours	381	54.8
3 - 6 hours	241	34.7
6 - 12 hours	67	9.6
More than 12 hours	6	0.9
How many hours per day of sitting are required from you in your job?		
Less than 3 hours	305	43.90
3 - 6 hours	274	39.40
6 - 12 hours	104	15.00
More than 12 hours	12	1.70
How many hours per day are standing or walking required in your daily activity (an hour per day)?		
Less than 3 hours	386	55.5
3 - 6 hours	203	29.2
6 - 12 hours	92	13.2
More than 12 hours	14	2
How many hours per day of prolonged sitting is required in your main daily activity (hours per day)?		
Less than 3 hours	340	48.90
3 - 6 hours	225	32.40
6 - 12 hours	103	14.80
More than 12 hours	27	3.90

**Figure 3:**
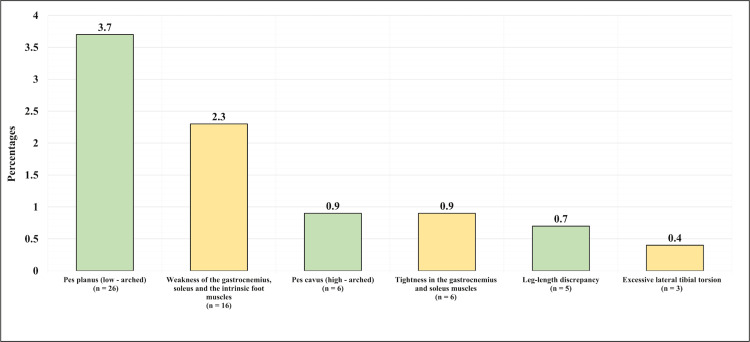
The history of lower limb conditions The figure has been created by the authors.

The most commonly reported conditions were as follows: pes planus (low-arched) in 26 (3.7%) of the participants; weakness of the gastrocnemius, soleus, and the intrinsic foot muscles; pes cavus (high-arched) in six (0.9%) of the participants; and tightness in the gastrocnemius, soleus and the intrinsic foot muscles in six (0.9%) of the participants (Figure 3).

**Figure 4:**
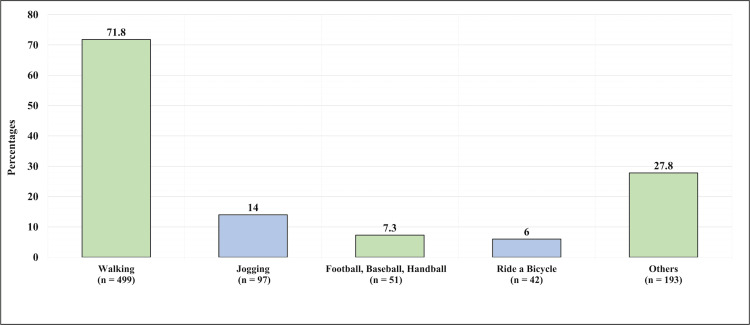
Exercises performed by the participants on a regular basis This figure has been created by the authors.

The most reported exercises were walking for 499 (71.8%), jogging for 97 (14%), and football, baseball, or handball for 51 (7.3%) participants, as illustrated in Figure 4.

Of the participants, 191 (27.5%) reported suffering from inferior heel pain on weight bearing or waking, while 504 (72.5%) reported that they did not suffer from any pain of that nature. Of the participants, 177 (25.5%) reported suffering from heel pain and tightness after standing up from bed in the morning or after they had been seated for a prolonged time, while 518 (74.5%) reported they did not feel this kind of pain. With regards to pain upon waking up in the morning, 134 (19.3%) reported having pain when rising in the morning, specifically in the plantar aspect of the foot, or sub calcaneal pain, while 561 (80.7%) did not experience it. Of the participants, 25 (3.6%) reported having been previously diagnosed with plantar fasciitis, while 670 (96.4%) were not. As for those who were diagnosed before, 10 (40%) had the right side affected, seven (28%) had the left side affected, and eight (32%) had both sides affected. With regards to treatment, 14 (56%) of those who were previously diagnosed had received anti-inflammatory agents as treatment, 10 (40%) received physiotherapy, four (16%) received local injections, three (12%) had foot orthosis, and one (4%) had surgical intervention, while four (16%) had not received treatment at all (Table 3).

**Table 3:** Plantar fasciitis profile of the participants (n=695)

Question	n	%
Do you suffer from inferior heel pain on weight bearing or waking?		
Yes	191	27.5
No	504	72.5
Do suffer from heel pain and tightness after standing up from bed in the morning or after you have been seated for a prolonged time?		
Yes	177	25.5
No	518	74.5
Do you have pain first thing after rising in the morning (plantar aspect of the foot, or sub calcaneal pain)?		
Yes	134	19.3
No	561	80.7
Have you ever been diagnosed with plantar fasciitis before?		
Yes	25	3.60
No	670	96.40
If you have been diagnosed with plantar fasciitis before, which side was affected? (n = 25)		
Right	10	40
Left	7	28
Both	8	32
After being diagnosed with plantar fasciitis, what kind of treatment did you receive?		
Anti-inflammatory agents (oral or local)	14	56.00
Foot orthosis	3	12.00
Physiotherapy (ultrasound, laser, exercises)	10	40.00
Local injections (steroid or plasma)	4	16.00
Surgical intervention	1	4.00
Nothing	4	16.00

**Figure 5:**
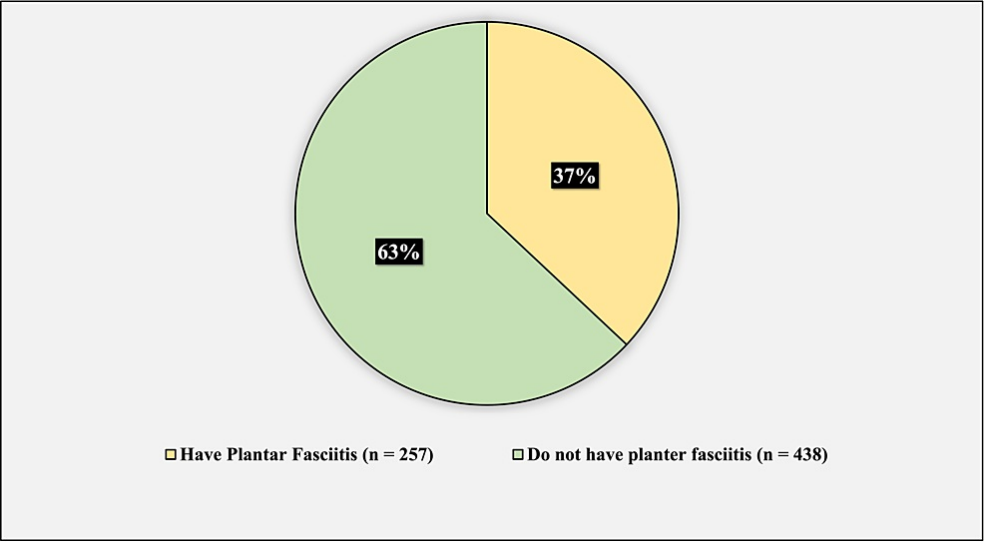
The prevalence of plantar fasciitis among the group of participants This figure has been created by the authors.

254 (37%) of the participants have plantar fasciitis, while 438 (63%) do not have plantar fasciitis (Figure 5).

The results of the Foot and Ankle Outcome Score (FAOS) survey are: the mean symptom subscale score was 57.81 + 11.28, the mean pain subscale score was 72.87 + 20.84, the mean daily living subscale score was 72.73 + 22.25, the mean sports and recreation subscale score was 76.83 + 23.06, and the mean quality of life subscale score was 70.23 + 25.17 (Table 4).

**Table 4:** The results of the Foot and Ankle Outcome Score (FAOS) survey (n = 257)

Symptoms subscale score		
Mean		57.81
Standard deviation		11.28
Pain subscale score		
Mean		72.87
Standard deviation		20.84
Daily living subscale score		
Mean		72.73
Standard deviation		22.25
Sports and recreation subscale score		
Mean		76.83
Standard deviation		23.06
Question	n	%
How often are you aware of your foot/ankle problem?		
Never	54	21.01
Monthly	95	36.96
Weekly	51	19.84
Daily	40	15.56
Constantly	17	6.61
Have you modified your lifestyle to avoid potentially damaging activities to your foot/ ankle?		
Not at all	146	56.81
Mildly	39	15.18
Moderately	37	14.40
Severely	13	5.06
Totally	22	8.56
How troubled are you with a lack of confidence in your foot/ankle?		
Not at all	115	44.75
Mildly	59	22.96
Moderately	39	15.18
Severely	22	8.56
Extremely	22	8.56
In general, how much difficulty do you have with your foot/ankle?		
Not at all	96	37.35
Mildly	78	30.35
Moderately	39	15.18
Severely	24	9.34
Extremely	20	7.78
Quality of life subscale score		
Mean		70.23
Standard deviation		25.17

Table 5 illustrates the univariate logistic regression (factors predicting plantar fasciitis). The univariate analysis included the following variables: age, gender, BMI; jogging, walking, riding a bike, playing (football, basketball, or handball); hours per day required on the job to be standing, walking, or sitting; hours per day required by daily activities to be standing, walking, or sitting.

The following factors predicted a higher rate of plantar fasciitis: being 40 to 55 years old (p < 0.001, odds ratio = 3.21), being 56 to 65 years old (p = 0.02, odds ratio = 3.84), being obese (p = 0.001, odds ratio = 2.97), having weakness in the gastrocnemius, soleus and the intrinsic foot muscles (p = 0.002, odds ratio = 7.73), exercising by playing (football, baseball, handball) (p = 0.004, odds ratio = 2.92), requiring a great amount of time standing or walking (p < 0.001, odds ratio = 2.96), jobs requiring a moderate amount of time standing or walking (p = 0.02, odds ratio = 1.96), jobs requiring the employee to stand or walk for three to six hours per day (p < 0.001, odds ratio = 1.91), and jobs requiring them to stand or walk for six to 12 hours per day (p = 0.02, odds ratio = 1.88). The following factors predicted a lower rate of plantar fasciitis: being male (p < 0.001, odds ratio = 0.57), and having pes planus (p = 0.011, odds ratio = 0.35).

**Table 5:** Univariate logistic regression (factors predicting the development of plantar fasciitis)

Univariate logistic regression (factors predicting having plantar fasciitis)					
Factor		P-Value	Odds Ratio	Confidence Interval	
Gender (male vs female)		< 0.001*	0.57	0.42	0.78
Age (less than 40 years is the referent)					
40 - 55 years		< 0.001*	3.21	2.25	4.59
56 - 65 years		0.02*	3.84	1.24	11.94
Older than 65 years		0.536	2.40	0.15	38.67
BMI (Underweight is the referent)					
Normal weight		0.586	0.85	0.47	1.54
Overweight		0.169	1.53	0.83	2.82
Obesity		0.001*	2.97	1.57	5.62
Having pes planus (low-arched) (yes vs no)		0.011*	0.35	0.16	0.79
Having weakness of the gastrocnemius, soleus, and the intrinsic foot muscles (yes vs no)		0.002*	7.73	2.18	27.38
Exercise (jogging) (yes vs no)		0.076	0.65	0.41	1.05
Exercise (walking) (yes vs no)		0.258	0.82	0.58	1.16
Exercise (ride a bicycle) (yes vs no)		0.405	1.33	0.68	2.61
Exercise (football, baseball, handball) (yes vs no)		0.004*	2.92	1.40	6.11
How much standing or walking is required from you by your job? (None is the referent)					
Great amount		< 0.001*	2.96	1.81	4.85
Moderate amount		0.002*	1.96	1.28	3.00
A little amount		0.331	0.77	0.45	1.31
Hours per day on the job to be standing or walking (less than three hours is the referent)					
3 - 6 hours		< 0.001*	1.91	1.37	2.67
6 - 12 hours		0.02*	1.88	1.11	3.18
More than 12 hours		0.309	2.31	0.46	11.63
Hours on the job to be sitting (less than three hours is the referent)					
3 - 6 hours		0.214	0.81	0.58	1.13
6 - 12 hours		0.571	0.88	0.55	1.39
More than 12 hours		0.315	0.51	0.14	1.91
Hours per day required by your daily activities to be standing or walking (less than three hours is the referent)					
3 - 6 hours		0.403	1.16	0.82	1.65
6 - 12 hours		0.107	1.46	0.92	2.32
More than 12 hours		0.238	1.90	0.65	5.54
Hours per day required by your daily activities to be sitting (less than three hours is the referent)					
3 - 6 hours		0.265	0.82	0.57	1.16
6 - 12 hours		0.605	1.13	0.72	1.77
More than 12 hours		0.773	1.13	0.51	2.50
* Significant at level 0.05					

Table 6 illustrates the multivariate logistic regression (factors predicting having plantar fasciitis). The multivariate analysis included the following variables: age, gender, BMI, having pes planus (low-arched), having weakness of the gastrocnemius, soleus, and intrinsic foot muscles, and the amount of time that the participants spend standing or walking for their jobs.

The following factors predicted a higher rate of planter fasciitis: being 40 to 55 years old (p < 0.001, odds ratio = 2.15), being 56 to 65 years old (p = 0.037, odds ratio = 3.58), being obese (p = 0.031, odds ratio = 2.16), having weakness in the gastrocnemius, soleus, and the intrinsic foot muscles (p = 0.003, odds ratio = 7.39), jobs requiring a great amount of time standing or walking (p < 0.001, odds ratio = 3.17), and jobs requiring a moderate amount of time standing or walking (p = 0.012, odds ratio = 1.83). Being male predicted a lower rate of plantar fasciitis (p < 0.001, odds ratio = 0.52).

**Table 6:** Multivariate logistic regression (factors predicting plantar fasciitis)

Factor		P-Value	Odds Ratio	Confidence Interval	
Gender (male vs female)		< 0.001*	0.52	0.37	0.74
Age (less than 40 years is the referent)					
40 - 55 years		< 0.001*	2.15	1.44	3.23
56 - 65 years		0.037*	3.58	1.08	11.89
Older than 65 years		0.831	1.36	0.08	22.85
BMI (underweight is the referent)					
Normal weight		0.475	0.79	0.42	1.49
Overweight		0.684	1.15	0.60	2.20
Obesity		0.031*	2.16	1.07	4.36
Having pes planus (low-arched) (yes vs no)		0.087	2.18	0.89	5.34
Having weakness of the gastrocnemius, soleus and the intrinsic foot muscles (yes vs no)		0.003*	7.39	1.97	27.78
How much standing or walking is required from you by your job? (none is the referent)					
Great amount		< 0.001*	3.17	1.84	5.48
Moderate amount		0.012*	1.83	1.14	2.93
A little amount		0.666	0.88	0.50	1.56
* Significant at level 0.05					

## Discussion

Assessing the prevalence and risk factors for plantar fasciitis is important as it affects patient comfort and quality of life. In particular, studying risk factors is also crucial as it is directly related to the efficiency of the proper intervention to reduce the incidence and prevalence of plantar fasciitis [17].

The main aim of the current study was to estimate the prevalence of plantar fasciitis and to assess the risk factors for the condition among the Jazan population.

In regards to the socio-demographic characteristics of the participants, more than two-thirds (72.9%) of the participants were younger than forty years old. About half (50.4%) of the population were males, and the other half (49.6%) of the participants were females. Concerning marital status, about more than half (54%) of the participants were married. The vast majority (82.2%) of the participants had university-level education. In regards to occupation, more than half (55%) of the participants were employees. Regarding the BMI of the participants, 8.9% of the participants were underweight, 43.3% of the participants had normal weight, 28.5% of the participants were overweight, and 19.3% of the participants were obese.

About 6.2% of the participants had hypertension, 5.3% of the participants had diabetes, 1.2% of the participants had cardiovascular disease, 4% of the participants reported tobacco use or had tobacco-related conditions, and similar findings were reported in the parallel study which was conducted by Morony et al., in which comorbidities were present in about 7% of the participants with plantar fasciitis [18].

Most (26.9%) of the participants were found to be working in teaching, followed by 17.3% of the participants who were found to be working in healthcare and the rest of them doing other jobs. More than one-third (39.3%) of the participants reported that their work required a moderate amount of standing or walking. About 19.9% of the participants reported that their work required a small amount of standing or walking, and slightly more than one-fifth (22.6%) of the participants reported that their work required no standing or walking. In the study carried out by Messing et al. [19], plantar fasciitis was reported to be caused by working for long hours walking or standing.

The most commonly reported lower limb conditions were: pes planus (low-arched) in 3.7% of the participants, and it was also reported to be associated with plantar fasciitis in the congruent study conducted by Young et al., and it also mentioned limb length discrepancy and over pronation as risk factors [20]. Weakness and tightness of the gastrocnemius soleus and the intrinsic foot muscles were found in 0.9% of the participants. Similar findings were reported in the study conducted by Huerta et al., in which tightness of the gastrocnemius muscle was found to be linked with plantar fasciitis in most of the participants [21].

The most reported exercises were walking, as reported by 71.8% of the participants; jogging, as reported by 14%; and football, baseball, or handball, mentioned by 7.3%. Exercises and running were reported in the study carried out by Taunton et al. to be one of the risks of developing plantar fasciitis, and another parallel study carried out by Sobhani et al. also reported dancing as a cause of plantar fasciitis [22,23].

More than half (56%) of those who had previously been diagnosed with plantar fasciitis received anti-inflammatory agents; two-fifths (40%) received physiotherapy; about 16% received a local injection; 12% had foot orthosis; and 4% had surgical intervention; while 16% had not received treatment. Similar findings were reported in the study conducted by Tim et al., in which analgesia and local therapeutic options were the most commonly used treatments for plantar fasciitis [24].

Among the participants, 37% were found to have plantar fasciitis, and this prevalence was found to be higher than that found in the study conducted by Thomas et al., in which a prevalence of 9.6% was reported, and this could be attributed to factors such as activities, work hours, and other factors [25].

Regarding the Foot and Ankle Outcome Score (FAOS) survey, the mean symptoms subscale score was found to be 57.81. The mean pain subscale score was found to be 72.87 and this was higher when compared to the study which was conducted by van den Akker-Scheek et al. in which a scale range of 33 to 83 was reported [26].

The mean quality of life subscale score was 70.23. The mean daily living subscale score was 72.73, and this was higher than that reported in the study carried out by Piva et al., in which the daily living score was found to be 63 [27].

Results of the univariate analysis showed that the following factors predicted a higher rate of plantar fasciitis: being 40 to 55 years old; being 56 to 65 years old; having weakness of the gastrocnemius, soleus, and intrinsic foot muscles; exercising by playing (football, baseball, handball); jobs requiring a great amount of time standing or walking; jobs requiring a moderate amount of time standing or walking; jobs requiring three to six hours per day of standing or walking; and jobs requiring six to 12 hours per day of standing or walking. The following factors predicted a lower rate of plantar fasciitis: being male and having pes planus. In regards to age, similar findings were reported in the study conducted by Dunn et al., in which middle-aged participants were the most affected by plantar fasciitis [28].

Multivariate analysis revealed that the following factors predicted a higher rate of plantar fasciitis: being 40 to 55 years old, being 56 to 65 years old, being obese, having weakness of the gastrocnemius, soleus, and intrinsic foot muscles, jobs requiring a great amount of time standing or walking, and jobs requiring a moderate amount of time standing or walking. Being male predicted a lower rate of plantar fasciitis and this was found to be contradictory to the study which was conducted by Granado et al. which found no difference between males and females in terms of developing plantar fasciitis [29].

Limitations

This study is a cross-sectional study in which the participants answered an online questionnaire. The lack of clinical examination and the variation in responses of the participants make this study limited.

## Conclusions

Plantar fasciitis pain is a prevalent disabling condition with considerable effects on quality of life. In this study, we found that long hours of work in walking or standing positions were associated with an increased risk of developing plantar fasciitis. Middle age, prolonged exercise, and gastrocnemius muscle tightness were also associated with the development of plantar fasciitis. Efforts should be directed towards health education of the population about the risk factors and management of plantar fasciitis.
